# First identification of proteins involved in motility of *Mycoplasma gallisepticum*

**DOI:** 10.1186/s13567-014-0099-2

**Published:** 2014-10-17

**Authors:** Ivana Indikova, Martin Vronka, Michael P Szostak

**Affiliations:** Department of Pathobiology, Institute of Bacteriology, Mycology and Hygiene, University of Veterinary Medicine Vienna, Veterinaerplatz 1, A-1210 Vienna, Austria

## Abstract

*Mycoplasma gallisepticum*, the most pathogenic mycoplasma in poultry, is able to glide over solid surfaces. Although this gliding motility was first observed in 1968, no specific protein has yet been shown to be involved in gliding. We examined *M. gallisepticum* strains and clonal variants for motility and found that the cytadherence proteins GapA and CrmA were required for gliding. Loss of GapA or CrmA resulted in the loss of motility and hemadsorption and led to drastic changes in the characteristic flask-shape of the cells. To identify further genes involved in motility, a transposon mutant library of *M. gallisepticum* was generated and screened for motility-deficient mutants, using a screening assay based on colony morphology. Motility-deficient mutants had transposon insertions in *gapA* and the neighbouring downstream gene *crmA.* In addition, insertions were seen in gene *mgc2,* immediately upstream of *gapA,* in two motility-deficient mutants. In contrast to the GapA/CrmA mutants, the *mgc2* motility mutants still possessed the ability to hemadsorb. Complementation of these mutants with a *mgc2-*hexahistidine fusion gene restored the motile phenotype. This is the first report assigning specific *M. gallisepticum* proteins to involvement in gliding motility.

**Electronic supplementary material:**

The online version of this article (doi:10.1186/s13567-014-0099-2) contains supplementary material, which is available to authorized users.

## Introduction

Motility is regarded as a virulence factor in many pathogenic bacteria. The ability to move enables microorganisms to reach a specific niche or to leave hostile environments. Amongst motile bacteria, various mechanisms to create a momentum have evolved. In *Bordetella bronchiseptica*, *Escherichia coli*, and *Salmonella enterica* serovar Typhimurium flagellar motility has been shown to be crucial for the initial stages of infection, while in *Legionella pneumophila* motility is necessary to establish and maintain infection [1]. In contrast to these species, in which motility can be downregulated to favor a specific life-style, some bacteria, such as *Helicobacter*, *Campylobacter*, and *Pseudomonas aeruginosa,* depend on constitutive flagellar motility for successful infection [1]. Experiments showing that only motile bacteria can be reisolated after infection with a mixed population of motile and non-motile variants underline the importance of motility in the infection process [2].

Mycoplasmas lack a cell wall and are considered to be the smallest self-replicating microorganisms. They have limited biosynthetic capabilities as they are highly adapted to a parasitic life-style [3]. In spite of the many limitations that have resulted from their degenerative evolution, some mycoplasmas have the ability to travel over inert surfaces, like glass, plastic or over eukaryotic cells, even though they lack any obvious locomotory appendages such as flagella or pili [4].

*Mycoplasma gallisepticum* is an avian pathogen causing chronic respiratory disease in chickens and infectious sinusitis in turkeys, that is known to possess gliding motility. Like the majority of gliding mycoplasmas, *M. gallisepticum* belongs to the *M. pneumoniae* cluster [5], named after *M. pneumoniae,* the causative agent of human bronchitis and atypical pneumonia [6]. The mechanism that enables *M. pneumoniae* and other mycoplasmas to glide has been the subject of a number of studies [7].

The best studied gliding mechanism is that of *M. mobile,* isolated from the gills of a fresh-water fish [8], which is phylogenetically distant from the *pneumoniae* cluster. *M. mobile* can be cultivated at room temperature and its average gliding speed is 2 to 4.5 μm/s [9], thus visualization of the gliding process is not dependent on additional microscope equipment such as a plate heater or a computer-connected CCD video camera. Several proteins of *M. mobile* have been identified as motility proteins [10]. Centered at the neck region of the jellyfish shape-like *M. mobile*, the Gli349 leg protein binds to sialylated oligosaccharides on glass or animal cells. Together with the Gli521 gear protein and the Gli123 mount protein, a large number of legs may act in a continuous “bind, pull, and release” mode, thereby creating a continuous pull in the forward direction. The multiple legs involved suggested the term “centipede-like” locomotion [11].

However, no homologs of these *M. mobile* motility genes have been found in *M. pneumoniae* or *M. gallisepticum*, indicating that different mycoplasmas may have developed different gliding machineries. The motile members of the *M. pneumoniae* cluster share a characteristic morphological feature, cellular polarity. These mycoplasmas have a flask-shaped appearance, strengthened by a cytoskeleton, and have a differentiated tip structure, often called the attachment tip or terminal organelle (TO). In *M. pneumoniae*, the TO mediates adherence to the host respiratory epithelium, a prerequisite for successful colonization [12]. In addition, the TO is the leading end in gliding motility [13], as cells always glide in the direction of the tip structure.

Formation of the TO appears to be a complex process that has to be well orchestrated, chronologically and spatially [14]. The TO of *M. pneumoniae* consists of a network of cytadherence proteins, including P1, P30, the accessory proteins P65, B, C, and the structural proteins HMW1, HMW2, and HMW3 [15]. Mutations affecting cytadherence or the correct assembly of the TO have direct effects on gliding motility. Loss of proteins P1, P30, or P65 lead to a non-motile, as well as hemadsorption-negative, phenotype [16]. Similarly, mutations in the TO proteins P41 and P24 have an impact on the velocity and frequency of gliding [17]. Although several elements of the gliding machinery have been identified, it is still unclear how these motility-associated proteins work in concert to generate a propulsive force and move the cell forward.

Studies to elucidate the motility mechanisms of members of the pneumoniae cluster have also included *M. genitalium,* a close relative of *M. pneumoniae.* Their proteins share a high degree of homology [18]. Many of the proteins involved in *M. pneumoniae* motility have counterparts in *M. genitalium* [19]. Surprisingly, no protein involved in motility has yet been identified in *M. gallisepticum*, and although *M. gallisepticum* was included in a recent study of mycoplasma gliding [20], little is known about the proteins involved. Therefore, we examined the gliding ability of *M. gallisepticum* strain R and clonal variants of it, including a library of transposon insertion mutants. The aim of this study was to identify proteins that contribute to the motility process of *M. gallisepticum*, to investigate the molecular properties of such motility proteins, and to further refine the tools for screening and complementing motility mutants.

## Materials and methods

### Strains and growth conditions

*M. gallisepticum* strains Rlow, Rhigh [21], RCL1, RCL2, mHAD3 [22], motility mutants and complemented motility mutants were cultured in modified Hayflick medium [23] (HFLX) at 37 °C. To grow tetracycline- (Tc^R^) or chloramphenicol- resistant (Cm^R^) *M. gallisepticum* transformants, either Tc (4 μg mL^−1^; Roche Diagnostics, Penzberg, Germany) or Cm (17 μg mL^−1^; Carl Roth GmbH & Co KG, Karlsruhe, Germany) were added to HFLX medium. *Escherichia coli* DH10B (Invitrogen Corp., Carlsbad, CA, USA) was used for the propagation of plasmids used in this study.

### Motility assays

To detect satellite growth of *M. gallisepticum*, a freshly grown culture was seeded in a 24-well microtiter plate at a concentration of 40 CFU per 400 μL of HFLX medium per well. After 2 h of attachment, the medium was replaced by HFLX containing 2% gelatin and, if transformants were to be analyzed, Tc was added to the HFLX-gelatin mixture. Colony morphology was examined after growth at 37 °C for five to seven days using an SMZ-U stereomicroscope (Nikon Corp., Tokyo, Japan).

Characterization of *M. gallisepticum* gliding motility was performed using a microcinematography motility assay (MMA). For this purpose, 100 μL of a culture freshly grown in HFLX medium was placed on a standard microscope glass slide (Thermo Fisher Scientific Inc., Waltham, MA, USA). After 1 h of incubation at 37 °C, attached cells were overlaid with 100 μL of fresh medium containing 2% gelatin. After 1.5 h of incubation, cell movement was examined using an Olympus AX70 microscope equipped with a heating plate set at 37 °C, and phase-contrast images were captured at 1-s intervals for a total of 180 s with a Color View CCD digital camera controlled using CellPlus (Olympus Soft Imaging Solutions GmbH, Muenster, Germany).

Computer-assisted qualitative analysis of motility was performed by overlaying 180 single frames of a 3 min microscope movie with the Z project tool of the Fiji image processing package [24], choosing “Minimum Intensity” as the critical parameter. Bacterial paths were highlighted by standard layer manipulations using Photoshop CS3 version 10.0 (Adobe Systems Inc., San Jose, CA, USA).

For a quantitative analysis of motility, the ten mycoplasmas with the longest Z project paths in each field of view were selected, and their movements were tracked using the ImageJ/Fiji MTrackJ plugin [25]. Using the analysis tool of MTrackJ, the distance travelled and the overall speed of motility, including resting periods, were determined. The best three results of 5 independent experiments were chosen for graphical representation.

Results of quantitative MMAs were analyzed for statistical significance by using a two-tailed Student’s *T* test [26]. *P* values of ≤ 0.05 were considered to indicate significant differences between groups.

### DNA isolation and sequencing reactions

Genomic DNA from mycoplasmas was isolated using the GenElute™ Mammalian Genomic DNA Miniprep Kit (Sigma-Aldrich Chemie GmbH, Taufkirchen, Germany). Plasmid DNA from *E. coli* cultures was purified using the PureYield™ Plasmid System Kit (Promega, Mannheim, Germany). Oligonucleotide synthesis was performed by either Microsynth (Microsynth AG, Balgach, Switzerland) or Invitrogen (Life Technologies GmbH, Darmstadt, Germany), and DNA sequencing was conducted by LGC Genomics (LGC Genomics GmbH, Berlin, Germany). If not otherwise mentioned, all enzymes used in this study were purchased from Promega. For DNA/PCR purification, the Wizard® SV Gel and PCR Clean-Up System (Promega) was used.

### Construction of plasmids

#### Transposon Tn*4001*cam

To use transposon mutants in gentamicin-based cell invasion assays, we first had to replace the gentamicin resistance gene of Tn*4001*. The chloramphenicol-resistance cassette Cm^R^ of plasmid pACYC184 (Invitrogen) was amplified using primers Xcat5 and Xcat3, introducing *Bam*HI and *Nar*I cleavage sites, respectively. The purified amplicon was cloned into the corresponding sites of plasmid p5TlacZ + [27], thereby placing the Cm^R^ cassette under the control of tuf*PO* in plasmid p5xCAT. Left (ISL) and right (ISR) IS256 elements of *S. aureus* transposon Tn*4001*mod were amplified from plasmid pISM2062 [28], using primers ISR-f and ISR-r, which introduced *Mlu*I and *Kas*I cleavage sites, and ISL-f and ISL-r, which contained *Sac*II and *Sal*I cleavage sites. The amplicons ISL and ISR were cloned into the corresponding sites to the right and to the left end, respectively, of the Cm^R^ cassette on plasmid p5xCAT. Transformants of *E. coli* DH10B were selected on Luria-Bertani agar containing Cm (30 μg/mL). Transposon mutants of *M. gallisepticum* were stable for at least 20 passages without Cm and no re-transposition or excision of Tn*4001*cam could be detected (data not shown).

#### Integration plasmid p5Hmgc

Tn*4001*mod on plasmid pISM2062 [28] was modified by adding a 6xHis-tag and a multiple cloning site: a 51-bp DNA fragment, created by annealing oligonucleotides HisC-f and HisC-r, was inserted between the *BamH*I and *Sma*I cleavage sites of pISM2062, resulting in plasmid pTnHis. The *M. gallisepticum* gene *mgc2* was then amplified by PCR using genomic DNA of strain Rlow as template and primers ISM-mgcF and ISM-mgcR, and subcloned into pTnHis using the *Bam*HI and *Sph*I cleavage sites. The resulting plasmid, pTHmgc, was linearized with *Not*I, treated with the Klenow fragment of DNA polymerase I (New England Biolabs GmbH, Frankfurt/Main, Germany) to fill in the 5′ overhang, and subsequently digested with *Bam*HI. A 1093-bp fragment was gel-purified and ligated to a 3.5-kb fragment of plasmid pINT [27], obtained after digestion with *Bam*HI and *Sfo*I.

#### Transformation of mycoplasmas

*M. gallisepticum* transposon mutants were generated by electroporation of strain RCL1 with 3–5 μg of pTnC, as described previously [22]. For the transformation with integration plasmid p5Hmgc, 20–30 μg of plasmid DNA was used. Following electroporation, mycoplasma cells were cultured on HFLX plates containing either 17.5 μg chloramphenicol mL^−1^ or 4 μg tetracycline mL^−1^*.*

#### Ligation mediated PCR (LM-PCR)

Transposon insertion sites were determined by LM-PCR using the method of Sharma et al. [29], with modifications. Briefly, genomic DNA of *M. gallisepticum* transposon mutants was digested with *Bgl*II, and ligated to the *Bgl*II-adaptors Ad1B and Ad2B. Prior to ligation, the adaptor oligo nucleotides were dissolved separately in double distilled water at a concentration of 100 μM and equal volumes of both were mixed together. The mixture was incubated at 70 °C for 10 min, allowed to cool gradually to 40 °C, and then incubated at 40 °C for 10 min. The mixture was then cooled gradually to 25 °C and stored frozen in small aliquots until further usage. The ligation product was used as a template for PCR amplification using the adaptor-specific primer Bgl and primer IS-I, specific for IS256 of Tn*4001*mod. The PCR product was then used as template for a semi-nested PCR using primers Bgl and IS-N, and the gel-purified amplicons were sequenced (Microsynth).

### Production of antibodies

#### MGC2

The full-length *mgc2* gene was amplified using the LR-PCR kit (Roche) and primers mgc2_3 and mgc2_4 (Table 1), introducing *Eco*RI and *Hin*dIII cleavage sites at either end. The TGA codon was mutagenized to TGG using primer mgc_tga and the Site-directed Mutagenesis kit (Stratagene) according to the manufacturer’s instructions. The mutated *mgc2* gene was then cloned between the *Eco*RI and *Hin*dIII cleavage sites in pRSET (Invitrogen) and introduced into *E. coli* BL21 (DE3)pLys Star (Invitrogen). Expression of MGC2 was induced by addition of 0.5 mM isopropyl β-D-1-thiogalactopyranoside (IPTG) and the protein was purified using the MagneHis™ Protein Purification System (Promega) and immobilized metal affinity chromatography using ProBond™ nickel-chelating resin (Invitrogen) according to the manufacturers’ instructions. Purity of the MGC2 protein was confirmed by Western blotting using Anti-Xpress™ antibodies (1:5000) (Invitrogen). After elution and dialysis against phosphate-buffered saline (PBS), the protein was used for immunization of rabbits as described elsewhere [30].

**Table 1:** **Oligonucleotides used in this study**

**Primer**	**Sequence (5′ to 3′)**	**Product (length [bp])**
**Ad1B**	CTCGTAGACTGCGTACC	LM-PCR (variable)
**Ad2B**	GATCGGTACGCAGTCTAC	
**C’gapA5**	ATTAggatccAGTATTCAACGTTTCTAAG	MG *gapA* (911 bp)
**C’gapA3**	TACGaagcttACCTTAATTATTCAATTTTC	
**HisC-f**	GATCCCTCGAGCCCGGGGCATGCCATCATCATCATCATCATTAATAGGG	synthetic 6xHis-tag
**HisC-r**	CGCCCTATTAATGATGATGATGATGATGGCATGCCCCGGGCTCGA	
**ISL-f**	TATAccgcggATAAAGTCCGTATAATTGTG	IS256L (1365 bp)
**ISL-r**	TATAccgcggATAAAGTCCGTATAATTGTG	
**ISR-f**	TATAacgcgtGATAAAGTCCGTATAATTGTG	IS256R (1342 bp)
**ISR-r**	ATTggcccgAAAATAATAAAGGAAGTGAGTC	
**ISM-mgcF**	ATAAggatccTGTTGAAAAGCGCTTAGC	MG *mgc2*
**ISM-mgcR**	TTAAgcatgcTCTAGGTCCATTTTGTGG	(1001)
**Bgl**	TAGACTGCGTACCGATC	LM-PCR
**IS-I**	TGTACCGTAAAAGGACTG	products
**IS-N**	AAAGGACTGTTATATGGC	(variable)
**mgc2_3**	ACGCAGgaattcATAACAATTATG	MG *mgc2*
**mgc2_4**	TTTACAaagcttGTCTTATCTAGG	(894)
**mgc_tga**	GAAAGATTACCTCCGAACCATGGTTTTATCCAGTAGTGGG	TGA > TGG
**Xcat5**	TAGATGggatccATGGAGAAAAAAATCACTG	pACYC184 (751)
**Xcat3**	ATAAATggcgccCGCTTATTATCACTTATTC	*Cm*PO *+ Cm* ^*R*^

#### C-GapA

The 3′-terminal part of the *gapA* gene was amplified using the LR-PCR system (Roche) and primers C’gapA5 and C’gapA3, introducing *Bam*HI and *Hin*dIII cleavage sites, at either end. The gel-purified amplicon was ligated into plasmid pRSET-B (Invitrogen) and the resulting plasmid was introduced into *E. coli* BL21 (DE3)pLys Star (Invitrogen). The recombinant culture was grown at 28 °C, and gene expression was induced by addition of 0.3 mM IPTG at the early logarithmic growth phase. A protein of 35 kDa was retrieved from a sodium dodecyl sulfate polyacrylamide (SDS-PAA) gel after negative staining with a zinc stain and destain kit (BioRad Laboratories Inc., Hercules, CA, USA) and electroelution in an Electro-Eluter Model 422 (BioRad). The immunization of rabbits with the purified C-terminal part of GapA followed exactly the same procedures as for MGC2 antibodies.

#### CrmA

The generation of CrmA-specific antibodies has been described previously [30].

#### Western blot analyses and tryptic digestion

Five millilitre aliquots of overnight cultures of mycoplasmas were centrifuged at 4200 *g* for 30 min, and the cell pellets washed once with PBS and resuspended in PBS containing 0.05% of a commercial trypsin/EDTA solution (Life Technologies, #15400). Control samples were treated in the same way but without trypsin. The samples were incubated at 37 °C, 1-mL samples removed after 10, 30, 40, 50, 70 and 90 min, and trypsin activity stopped by adding phenylmethylsulfonyl fluoride (PMSF) at a final concentration of 1 mM. Cells were then collected by centrifugation and the presence of MGC2 was assessed by Western blot analysis. The solubilization and separation of mycoplasma cell lysates using 10% SDS-PAA gel electrophoresis, and Western blot analysis is described elsewhere [31]. Membranes were probed with antibodies against MGC2 (1:500), GapA (1:6000), or CrmA (1:2000) using peroxidase-conjugated swine-anti-rabbit IgG (1:2000, Dako) as secondary antibody, or with anti-6xHis antibody (1:6000, Aviva Systems Biology Corp., San Diego, CA, USA) in combination with the AffiniPure Goat Anti-Mouse IgG, Fc Fragment specific (1:10 000, Jackson ImmunoResearch Laboratories, Inc., West Grove, PA, USA).

#### Hemadsorption assay

The ability of mycoplasma colonies grown on HFLX agar plates to hemadsorb was tested as described previously [30].

#### Scanning electron microscopy (SEM)

Mycoplasma samples for SEM were prepared as described previously [32], except that mycoplasma cultures were grown at 37 °C on glass coverslips, precoated with poly-L-lysine (Sigma-Aldrich) according to the manufacturer’s instructions.

## Results

### Identification of non-motile *M. gallisepticum* strains

**Figure 1:**
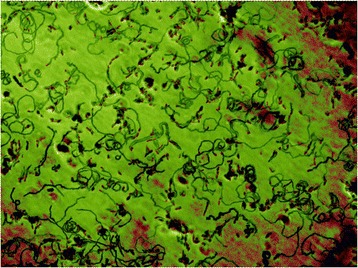
**Gliding paths of motile** ***M. gallisepticum.*** A stack of phase-contrast pictures of *M. gallisepticum* RCL1, captured at 1-s intervals, was manipulated with Fiji by applying a Z projection method [24] to visualize the paths of gliding mycoplasmas. Differential colouring of mycoplasmas (red) and their paths (green) was done with Adobe Photoshop. This qualitative microcinematography motility assay allowed the rapid assessment of the motility of wild-type and mutant strains.

To establish a method for screening for motility mutants of *M. gallisepticum*, we first identified motile and non-motile strains in our culture collection. Gliding of *M. mobile* and *M. pneumoniae* depends on cytadherence-associated components [16,33,34]. Therefore, we analyzed selected strains of *M. gallisepticum* that differed in hemadsorption (HA) (Table 2) using a qualitative microcinematography motility assay (MMA). The HA-positive (HA^+^) *M. gallisepticum* strain Rlow and its clonal variant RCL1, expressing the major cytadherence gene *gapA* as well as the cytadherence-related gene *crmA*, were capable of gliding. At any time, 60% of cells were moving, interrupted by short resting periods. Mycoplasma gliding paths were visualized by computer-generated overlay of all frames of a 3 min video and consisted mainly of circles and bends (Figure 1). In contrast, no gliding could be visualized for strains Rhigh, RCL2, or mHAD3, which lack either GapA and/or CrmA. These HA^−^ strains appeared to have lost the ability to glide, as no moving cells were observed in MMAs, in spite of numerous trials under a variety of conditions.

**Table 2:** **Protein content, motility and hemadsorption ability of MG strain**

**Strain**	**Hem-adsorption**	**Motility**	**Cell Shape**	**Presence of**			**Reference**
				**MGC2**	**GapA**	**CrmA**	
Rlow	**++**	**++**	flask	**+**	**+**	**+**	[21]
Rhigh	**-**	**-**	round	**+**	-	-	[21]
RCL1	**++**	**++**	flask	**+**	**+**	**+**	[22]
RCL2	**-**	**-**	round	**+**	-	-	[22]
mHAD3	**-**	**-**	round	**+**	(+)	-	[22]
T932A	**+**	**-**	rounded flask	-	**+**	**+**	this study
T932C	**+**	**-**	distorted flask	trc^1^	**+**	**+**	this study

^1^trc; truncated.

### Colonies of motile *M. gallisepticum* form microsatellites

**Figure 2:**
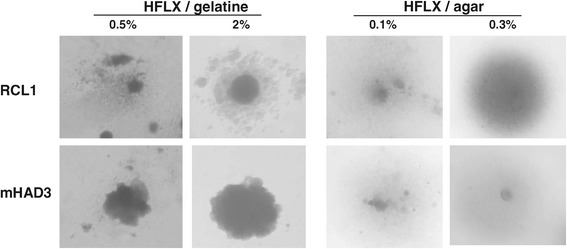
**Morphology of** ***M. gallisepticum*** **colonies.** Cultures of RCL1 and mHAD3 were either grown in HFLX medium solidified with 0.5 or 2% gelatin or on low-agar plates containing 0.1 or 0.3% agar. In gelatin-containing medium, the non-motile strain mHAD3 formed compact colonies, in contrast to the motile RCL1 which formed colonies surrounded by microsatellites.

Formation of microsatellites around colonies grown on agar plates has been described for *M. mobile* [33] and *M. pneumoniae* [16], and appears to be an indicator of gliding motility in mycoplasmas. *M. gallisepticum* colonies have been reported to grow without satellite formation under the conditions that allow *M. mobile* to form microsatellites [33]. Conditions for microsatellite formation by *M. gallisepticum* were established using strains Rlow and Rhigh as prototypes of motile and non-motile strains (Table 2). Diffuse colonies spreading in all directions were observed for both strains when grown on HFLX medium solidified with a range of concentrations of agar (0.05 - 0.3%) (Figure 2). Higher concentrations of agarose have already been shown to allow only the formation of the typical fried-egg colonies and were therefore not tested.

When *M. gallisepticum* cells were first allowed to attach to the surface of a cell culture dish and then overlaid with HFLX medium containing gelatin, motile strains could be differentiated from non-motile strains (Figure 2). At 0.5% gelatin, Rlow and Rhigh were not able to form colonies on the bottom of the dish, but cloudy regions in the overlay medium indicated that mycoplasma cells had spread throughout the medium. With increasing gelatin concentrations, the number of colonies increased, while growth in the overlay medium decreased. At 2% gelatin, the motile strains Rlow and RCL1 formed round colonies with a smooth surface, surrounded by many satellites, while the non-motile strains Rhigh, RCL2, and mHAD3 formed colonies with a rough surface, uneven edges, and without satellite colonies. Higher concentrations of gelatin resulted in partial detachment of colonies and formation of microcolonies in the overlay medium. Therefore, for further experiments to detect colonies of *M. gallisepticum* with a satellite-growth altered (SGA) phenotype, HFLX medium solidified with 2% gelatin was used.

### Generation of motility-deficient mutants and complementation

**Figure 3:**
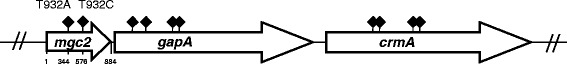
**Insertion sites of Tn** ***4001*** **cam transposon in motility mutants.** Non-motile RCL1 mutants harbored transposons in the *mgc* cytadherence locus, consisting of *mgc2*, *gapA* (formerly *mgc1*), and *crmA* (formerly *mgc3*). In mutants T932A and T932C Tn*4001*cam integrated 344 and 576 bp after the translational initiation nucleotide of *mgc2*.

To identify proteins involved in the gliding motility of *M. gallisepticum*, RCL1 was transformed with transposon Tn*4001*cam and transformed colonies were screened for the SGA phenotype. In a proof-of-concept study, 4000 colonies were screened and 38 mutants were found to exhibit defects in satellite colony formation. Their ability to glide was further examined individually using MMAs. Eight mutants had a low proportion of motile cells (20%), so they were stored for later analysis. Southern blot analyses of thirty motility mutants found that the majority carried multiple Tn*4001*cam insertions within their genome. Only ten mutants had contained only one or two transposon insertions. They were subjected to LM-PCR to precisely determine the transposon insertion sites. In four mutants the transposon had integrated into the *gapA* gene, and in another four mutants Tn*4001*cam was inserted into the *crmA* gene (Figure 3). These mutants were not analyzed further, because the same genes were affected in the cytadherence-negative, non-motile strains Rhigh, RCL2, and mHAD3. However, in two motility-deficient mutants the transposon had integrated into different sites in the *mgc2* gene (also known as the MGA_0932 coding region) and these mutant strains were designated T932A and T932C (Figure 3). After filter cloning, a LM-PCR analysis revealed that T932A contained the Tn*4001*cam at position 222 517 (numbering according to GenBank Accession AE015450.2) of the genomic DNA, 344 bp downstream of the initial coding nucleotide of the *mgc2* gene (Figure 3). The ORF therefore terminated 119 amino acids (aa) after the start codon, whereby the last 5 aa were encoded by the transposon. Even after repeated filter cloning mutant T932C still appeared to contain two transposons, one within the *mgc2* gene at position 222 749, which would allow for translation of 193 aa of the MGC2 protein. The second transposon was found in the CRISPR region at position 930 903.

To confirm that disruption of the *mgc2* ORF by transposition was responsible for the loss of motility, mutants T932A and T932C were complemented with an *mgc2-6xHis* fusion gene. For this purpose, the fusion gene was subcloned into a derivative of plasmid pINT [27], which integrates into the *oriC* region of *M. gallisepticum* by homologous recombination. The resulting plasmid p5Hmgc was introduced into the mutants by electroporation, and the integration into the genomic *oriC* locus was proven by Southern blot analyses.

### Characterization of *mgc2* mutants and complemented mutants

#### Expression and surface localization of MGC2

**Figure 4:**
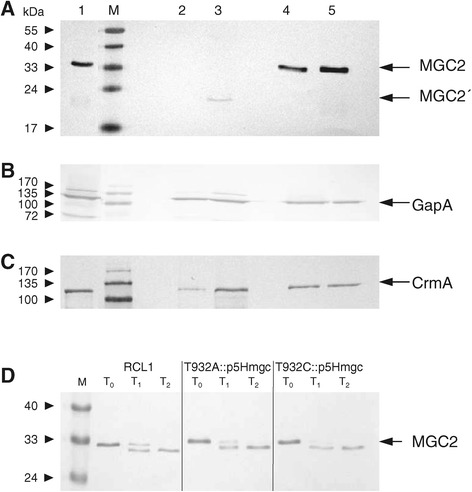
**Absence of MGC2 protein in gliding mutants.** Immunoblot analysis of RCL1 (lane 1), motility mutants T932A (lane 2) and T932C (lane 3), and complemented mutants T932A::p5Hmgc (lane 4) and T932C::p5Hmgc (lane 5) with antibodies specific for MGC2 **(A)**, GapA **(B)**, and CrmA **(C)**. No MGC2 was detected in T932A, and T932C weakly displayed a truncated MGC2′, while RCL1 and complemented mutants produced full-length MGC2. Immunoblot analysis of whole cell lysates of RCL1 and *mgc2*-complemented mutants T932A::p5Hmgc and T932C::p5Hmgc **(D)** after tryptic digest for 0 (T0), 40 (T1), and 90 min (T2); M, molecular weight marker.

The effect of transposon integration on expression of *mgc2* and the *gapA* and *crmA* genes immediately downstream of it was investigated by Western blot analyses. For this purpose, polyvalent rabbit antisera against MGC2, GapA and CrmA were produced. MGC2 was equally well detected in HA^+^ and HA^−^*M. gallisepticum* strains (Table 2). In contrast, no MGC2 could be detected in T932A lysates (Figure 4A, lane 2), and only a truncated MGC2, with an apparent size of 19 kDa, was detected in T932C (Figure 4A, lane 3). When complemented with p5Hmgc, both T932A and T932C expressed full-length MGC2 at concentrations comparable to RCL1 (Figure 4A, lanes 4–5). The C-terminal 6xHis-tag did not appear to influence the stability of the recombinant MGC2.

As it has been suggested that the *gapA* transcript initiates in the 3′ region of *mgc2* [35], we analyzed the expression of *gapA* and *crmA*. Western blot analyses revealed that expression of these genes did not appear to be affected by the Tn*4001*cam insertion into *mgc2,* as GapA and CrmA were detected at wild-type levels in both the *mgc2* mutants (Figures 4B and 4C).

MGC2 has been identified by immunoelectron microscopy on the mycoplasma cell surface [36]. To confirm this finding and to assess the size of the surface-exposed portion of MGC2, whole *M. gallisepticum* RCL1 cells were incubated with trypsin for different time periods and subjected to Western blot analyses with anti-MGC2 antiserum. Tryptic digestion produced a shorter fragment of MGC2, migrating at an apparent molecular mass of 31 kDa (Figure 4D), while untreated MGC2 migrated at 33 kDa. The 31-kDa band was first detected after 10 min of trypsin treatment (not shown), and became more prominent with longer periods of digestion. After 90 min of trypsin digestion the 33-kDa protein was no longer detectable (Figure 4D), indicating the degree of surface accessibility of MGC2 to trypsin. The fact that trypsin digestion reduced the molecular weight of MGC2 by only 2 kDa suggested that only a small region of MGC2 was exposed on the surface. Trypsin digestion patterns for the *mgc2*-complemented mutants T932A::p5Hmgc and T932C::p5Hmgc were comparable to those seen with RCL1 (Figure 4D), indicating the same degree of surface localization of the 6xHis-tagged MGC2 protein.

#### Hemadsorption activity

The hemadsorptive activity of wild-type strains, and cytadherence and motility mutants was analyzed using a standard HA assay. In contrast to the non-motile strains Rhigh, RCL2 and mHAD3, which have previously been reported to be deficient in cytadherence [22], the colonies of the motility-impaired *mgc2* mutants were able to bind erythrocytes and thus these strains were HA^+^ (Table 2 and Additional file 1).

#### Cell morphology

**Figure 5:**
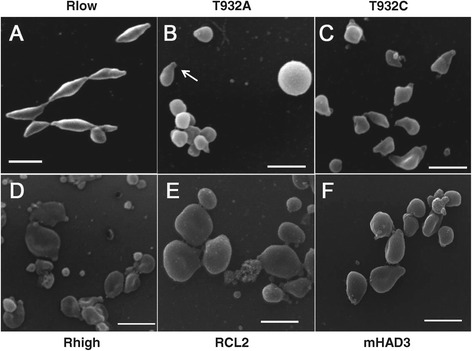
**Morphology of** ***M. gallisepticum*** **strains.** Scanning electron microscopy revealed the typical *M. gallisepticum* flask-shape morphology with a pronounced TO (**A** *,* Rlow), while CrmA- (**F**, mHAD3) or GapA-deficient strains (**D**, Rhigh; **E**, RCL2) tended to have an enlarged, round-shaped appearance with a less defined TO. Loss of MGC2 (**B**, T932A) or the presence of a truncated MGC2 (**C**, T932C) resulted in an overall smaller appearance with either a rounded body or a flask shape-like morphology with bulges and distortions. Scale bars, 1 μm.

Scanning electron microscopy revealed that the typical flask shape of the wild-type strain Rlow (Figure 5A) was altered in the non-motile, GapA and CrmA-lacking strains Rhigh, RCL2 and mHAD3, which consisted mainly of enlarged, rounded cells (Figures 5D-F). The *mgc2* mutant T932A appeared to have a smaller, coccoid morphology (Figure 5B). However, flask-shaped T932A cells with a slightly swollen body, but otherwise a well-defined TO, were also present (Figure 5B, arrow and Additional file 2). T932C had a wild-type flask shape except for a distorted bulging middle region (Figure 5C). For both mutants large spheroidal cells were also observed. The TOs of the more rounded GapA/CrmA mutants were generally not as well defined as in Rlow and T932C.

#### Gliding motility

Motility of T932A and T932C was assessed in at least 5 independent MMAs. Qualitative MMAs demonstrated the impaired motility of the *mgc2* mutants (Additional file 3) compared to RCL1. In contrast to the large number of long gliding tracks seen with RCL1 (Figure 1), only a very small proportion of the *mgc2* mutants produced gliding paths and these were very short. When complemented with *mgc2-*6xHis*,* these mutants were able to glide again over long distances, comparable in number and length to those of RCL1 (Additional file 3).

**Figure 6:**
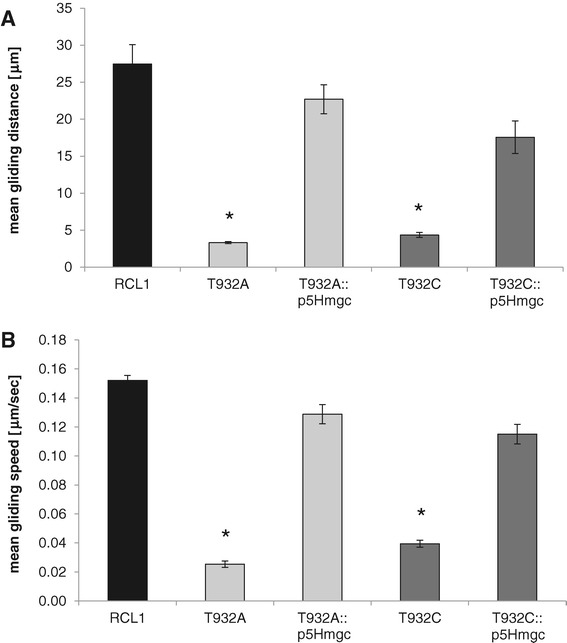
**Quantitative analysis of gliding motility.** Time-lapse cinematography was performed for cultures of parental strain RCL1 and the gliding-deficient transposon mutants (T932A, T932C) and *mgc2*-complemented mutants (T932A::p5Hmgc, T932C::p5Hmgc). Using the image processing Fiji package, movies were analyzed to determine the gliding distance **(A)** and the average speed **(B)** of mycoplasmas. The mean values from three independent experiments are shown. Statistically significant differences to RCL1 (*P ≤* 0.05) are marked by asterisks.

A quantitative MMA revealed that the mean gliding distance for wild-type *M. gallisepticum* RCL1 was about 27.5 μm over a 3 min observation interval. The gliding distances of the mutants T932A and T932C were significantly reduced to 12.1 and 15.9% of this, respectively (Figure 6A). The complemented strain T932A::p5Hmgc reached 82.7% of the gliding distance of RCL1, while T932C::p5Hmgc reached 63.9% of that of RCL1 (Figure 6A). Statistical analysis revealed that the mean gliding distance of complemented mutants was not significantly different from that of RCL1.

A similar relationship was seen when the mean gliding velocity over the same time interval was calculated. RCL1 glided with a mean speed of 150 nm s^−1^. The mutants T932A and T932C had a mean gliding velocity that was significantly reduced to 16.7 and 25.9% of that of RCL1, respectively, while *mgc2-*complementation restored the mean velocity of the mutants back to 84.6 and 75.6% of that of RCL1 (Figure 6B).

## Discussion

Not all bacteria are able to move. However, motile bacteria have a competitive advantage over their sessile relatives: motility enables bacteria to reach and remain in individual niches where they may find nutrients and/or shelter from the host’s defense mechanisms. Various motility mechanisms have evolved to allow bacteria to swim or float through liquid media, or to swarm, crawl, twitch or glide over solid surfaces. Many phylogenetically unrelated bacteria have been shown to be able to glide, some, like *Neisseria* and *Pseudomonas*, use surface appendices, while others, like *Flavobacterium* and *Myxococcus,* glide without any obvious locomotive structures [4]*.* Mycoplasmas are capable of gliding as well. In spite of the degenerative evolution process which shaped the *Mycoplasma* genomes to a minimum size, gliding motility seems to be essential for the parasitic life-style of some mycoplasmas. Of the currently described 132 *Mycoplasma* species [37], 14 motile species are listed: *M. agassizii*, *M. amphoriforme*, *M. gallisepticum*, *M. genitalium*, *M. imitans*, *M. insons*, *M. iowae*, *M. mobile*, *M. penetrans*, *M. pirum*, *M. pneumoniae*, *M. pulmonis*, *M. testudineum*, and *M. testudinis*. Interestingly, most of these mycoplasmas were either originally isolated from the human or animal respiratory tract, or they were at least occasionally recovered from such samples. As the respiratory tract is well protected against incoming particles by a thick layer of mucus and underlying epithelial cells covered with constantly beating cilia, gliding motility might be essential to overcome this mechanical barrier. The human pathogen *M. pneumoniae* has been shown to bind initially to the apical surface of ciliated human bronchial epithelial cells in vitro, then to move down towards the base of ciliated cells before spreading [38]. *M. pneumoniae* mutants that are defective in motility, but not in cytadherence, have impaired capacity to colonize differentiated bronchial epithelium in vitro [39], and these mutants cannot be recovered from the lung tissue four days after inoculation, whereas motile strains can be [40]. Gliding motility, therefore, seems to be essential for spreading of this pathogen in the respiratory tract, a first step in successful colonization of the host.

Contributing to mycoplasma pathogenesis, and being at the same time a possible target for the development of antimycoplasmal drugs, the elucidation of the mycoplasma motility mechanism is of major importance. Even though the gliding ability of the avian pathogen *M. gallisepticum* was already observed in the 1970s [13], little was known about the molecular basis of its motility. Here, we report for the first time the involvement of three proteins, MGC2, GapA and CrmA in the gliding motility of *M. gallisepticum.*

Both, GapA and CrmA have been shown before to be essential for cytadherence, colonization of the chicken trachea and induction of host responses [41-43], while *mgc2* has mainly been used to differentiate *M. gallisepticum* strains [44]. When *M. gallisepticum* strains from our culture collection were analyzed for motility, *gapA* mutants such as Rhigh and RCL2 were found to be non-motile. The finding that GapA is involved in the gliding mechanism of *M. gallisepticum* concords with data obtained for *M. pneumoniae.* Addition of a monoclonal antibody against the major cytadhesin of *M. pneumoniae*, P1, a homolog of GapA, removed gliding cells from the glass, but did not interfere with the binding of non-moving cells [34]. These data suggest that P1 has a crucial role in gliding motility of *M. pneumoniae,* independent of its adhesion properties, but that adhesion is a prerequisite for gliding [16,33,34,45]. Hasselbring et al. found that all motility and HA mutant strains of *M. pneumoniae* were able to bind to a glass surface [16]. Current models of the motility mechanism of *M. pneumoniae* suggest that P1 might serve as leg proteins that attach to sialylated oligosaccharides on glass or animal cells [46,47]. After binding to a solid support, with the energy provided by ATP hydrolysis, the legs might repeatedly bind, pull, and release surface structures, thus generating a continuous drag force that propels the cell forward. Addition of free sialylated oligosaccharides inhibits the motility of *M. pneumoniae* cells, but not the binding of non-gliding cells [47]. In our study, we similarly observed that GapA-deficient strains of *M. gallisepticum* lacked hemadsorption and motility, but were still able to attach to glass (data not shown), indicating that *M. gallisepticum* might be equipped with adhesion molecules of differently functionality.

*M. gallisepticum* strain mHAD3, which is HA^−^ and non-motile, carries a transposon in *crmA*, which lies directly downstream of *gapA*. The involvement of CrmA in motility is in concord with findings on *M. pneumoniae*, in which the *orf6* gene, a homolog of *crmA* [48], has been shown to be involved in motility. Mutant III-4, which lacks the ORF6 cleavage products P40 and P90, is non-motile [16]. Interestingly, P40 and P90 have been shown to complex with P1 in chemical cross-linking studies [49] and purified P1 and P90 have been found to form complexes in vitro [50], suggesting that P1 physically interacts with P90 in the mycoplasma membrane. Similarly, the *M. genitalium* proteins P110 and P140, homologs of GapA and CrmA, have been shown to be cytadhesins required for TO development and to be reciprocally dependent on each other for posttranslational stability [51]. Such mutual dependence has also been reported for GapA and CrmA in *M. gallisepticum* [22]. Mutations in *gapA* seem to have a polar effect on expression of *crmA*, as no CrmA is found in Rhigh or RCL2 [22,43]. A reason for this might be the operon structure of *gapA*/*crmA* (previously known as *mgc1*/*mgc3*) as suggested by Keeler et al., who mapped the transcriptional start site for these genes to the end of upstream *mgc2* [35]. However, loss of CrmA has a direct negative impact on the level of GapA. In mHAD3, the amount of GapA produced was greatly reduced [22], possibly a result from accelerated turnover of GapA due to the absence of its binding partner CrmA. However, a mutual dependence of GapA/CrmA is not certain, because some transposon mutants in *crmA* were reported to express GapA [41,52]. On the other hand, these transposon mutants were not analyzed for C-terminally truncated CrmA fragments, which might be sufficient to stabilize GapA. Knock-out of either *gapA* or *crmA*, therefore, might have an impact on expression of both proteins, and, as a consequence, it might be difficult to dissect whether the loss of motility in Rhigh, RCL2 or mHAD3 is attributable to the loss of GapA or CrmA.

To add another level of complexity, mutations in *gapA* or *crmA* have been shown to affect the morphology of *M. gallisepticum*. Our electron microscopy studies revealed that the typical flask-shaped appearance of *M. gallisepticum*, presenting a defined single knob-like structure at one polar end, changed to a rounder, bulkier morphology, with less defined tip structures in strains lacking GapA and CrmA. Similarly, *M. pneumoniae* mutant M5 which had lost the homolog of CrmA exhibits a perfect round cell shape, but has lost the tip-like structure [53]. Mutants of *M. pneumoniae* lacking homologs of GapA and CrmA also have lost the elongated flask-shape and display a branched cell morphology [54]. Our findings support a direct link between the major adhesin and the gliding mechanism. However, the question remains whether loss of GapA leads to a loss of motility because GapA cannot act any longer as the “leg” adhesin for the “bind-and-release”-cycles of gliding, or because loss of GapA leads to a drastically changed morphology with conceivable consequences on the correct positioning of any locomotive regions. As the correct morphology may be a strict requirement for the gliding process, future work should focus on the creation of defined *M. gallisepticum* mutants with modified variants of GapA or CrmA that have no mutual interference on expression of each other and a defined effect on only morphology, cytadherence, or motility.

To identify other genes involved in motility, we constructed a transposon by exchanging the gentamicin resistance gene in Tn*4001*mod [28] with the gene for chloramphenicol resistance, envisaging future needs such as assessing transposon mutants in gentamicin-based cell invasion assays. The chloramphenicol resistance gene of plasmid pACYC184 was effective when placed behind the MG *tuf*PO, which has previously been shown to function as an effective transcriptional promoter [27]. Stability assays showed that transposon mutants of *M. gallisepticum* could be cultivated for 20 passages without antibiotic selection pressure. No re-transposition or excision of Tn*4001*cam could be detected (data not shown). However, a drawback of this, and presumably any Tn*4001*-based transposon strategy, was the transposon’s tendency to integrate into multiple genomic sites simultaneously. Around 60% of our mutants were not further analyzed because they carried multiple insertions of Tn*4001*cam. The limited number of mutants analyzed in this study suggests that other genes involved in motility might be identified using a similar, more optimized approach. Recently, a mariner-based transposon was reported to produce mutants in *M. hyopneumoniae* with stable single insertions [55]. Use of a similar transposon would ensure that each mutant could be used for analysis.

Screening of a small transposon mutant library led to the identification of *mgc2* as a major motility gene. Loss of MGC2 resulted in a drastic reduction in motility (Figure 6) that could be restored by complementation of the mutants with a recombinant *mgc2*-6xHis gene. In contrast to the non-motile *gapA*/*crmA* mutants Rhigh, RCL2 and mHAD3, the *mgc2* mutation in T932A or T932C did not influence the presence of GapA or CrmA, and the cellular morphology was not as drastically altered as in the GapA/CrmA-deficient mutants. After carefully analyzing many electron micrographs, it seems that both *mgc2* mutants had a flask-shaped morphology similar to that of RCL1, characterized by the presence of a short TO (Figure 5). The majority of T932A cells appeared as coccoid cells, possibly a consequence of T932A binding almost exclusively via the TO to the glass slide surface. The flask-shaped T932A is then viewed along its longitudinal axis, with the distal end of the body orientated to the viewer and only virtually pretending a coccoid morphology. Occasionally, a TO became visible below the spherical body, bent towards the glass surface (Additional files 2A, B; indicated by arrows).

The ability to cytadhere, as measured by the HA assay, was not affected by the loss of MGC2 in T932A or T932C. This is in contrast to the first report about MGC2, which was classified as a cytadhesin [36], primarily based on the strong homology between MGC2 and the *M. pneumoniae* cytadhesin P30, and on attachment inhibition assays. Composed of an N-terminal domain I which is likely to be localized in the cytoplasm, a transmembrane region, a surface-exposed domain II, and highly repetitive, proline-rich domain III [56], P30 has been shown to be a membrane protein that co-localizes with the major cytadhesin P1 to the TO of *M. pneumoniae* [57]. MGC2 shares with P30 the same overall domain architecture [36,56], a similar size, and 40.9% amino acid sequence identity [36]. Specifically the transmembrane region and domain II are highly conserved between *M. pneumoniae* P30 and *M. gallisepticum* MGC2 [56]. Another coincidence is that P30 mutations have effects on cell morphology, cytadherence, motility and virulence [46]. The P30 null mutant II-3 has an ovoid, branched shape, has no ability to hemadsorb [58] nor glide [16], although all other cytadherence-related proteins, such as P1 and the cytadherence accessory proteins, are synthesized as usual [59]. Complementation of the II-3 mutant with the gene encoding P30 rescued the wild-type phenotype [46]. P30 mutations have an impact on the stability of P65 which is located at the distal end of the TO. P65 is involved in cytadherence and motility, and is thought to form a complex with P30 [60]. It would be interesting to investigate whether the reported reciprocal requirement for stabilization between P65 and P30 also exists between MGC2 and PlpA, the ortholog of P65 in *M. gallisepticum*.

A striking difference between P30 and MGC2 is its impact on cytadherence. In contrast to the HA^−^ P30 mutants of *M. pneumoniae,* the MGC2-deficient *M. gallisepticum* mutants were still able to hemadsorb. The binding of erythrocytes seemed to primarily depend on the presence of GapA or CrmA (Table 2). As the *mgc2* mutations did not have any polar effects on the expression of *gapA* or *crmA*, there was no influence on hemadsorption. However, the first description of MGC2 reported that MGC2-specific antiserum was able to reduce the attachment of *M. gallisepticum* to CEF cells by 30 to 48% [36]. The lack of complete inhibition was attributed to the existence of additional adhesins such as hemagglutinin VhlA (pMGA) or GapA (MGC1). Although HA has been widely used as an assay for cytadherence [3], the inhibition of attachment to CEF cells by MGC2-specific antibodies might not necessarily prove that MGC2 is a cytadhesin. This protein, as shown by our trypsinization assays, has a very small extracellular domain, and we hypothesize that MGC2 is rather linking internal components of the locomotive machinery to another external adhesion component. GapA could be the external adhesion component in analogy to the proposed leg protein P1 of *M. pneumoniae* [47], and antibody against MGC2 might affect GapA functions due to the close connection between MGC2 and the putative leg protein, thus causing only moderate inhibition of attachment.

We have shown the genes of the *mgc* locus [35], *mgc2*, *gapA*, and *crmA,* to be involved in motility of *M. gallisepticum*. It is of particular importance that *mgc2* is not involved in hemadsorption. This will enable us to study these two mechanisms, hemadsorption and motility, independently in *M. gallisepticum*.

## Additional files

Additional file 1:
**Qualitative HA assessment of wild-type and gliding mutants.** Colonies of RCL1 and motility mutants T932A and T932C were grown on agar plates and overlaid with sheep erythrocytes. No remarkable difference in hemadsorption was seen between wild-type RCL1 and *mgc2* mutant strains. Additional file 2:
**SEM pictures of** ***M. gallisepticum*** **motility mutants.** Mutant T932A **(A, B)** partly appeared as small spheres without a TO. In some cases TOs seemed to be placed between the glass surface and the main body of the mycoplasma cell (arrows), indicating that T932A had attached to the glass via the TO, therefore appearing spherical. Interestingly, in T932C **(C)** large spheroid cells with TO structures were also seen (triangle). Additional file 3:
**Gliding paths of** ***M. gallisepticum*** **motility mutants before and after complementation.** Only short gliding paths were seen, if any, in *mgc2* motility mutants (T932A and T932C), while the complementation of the mutants with *mgc2* (T932A::p5Hmgc and T932C::p5Hmgc) restored the gliding motility to almost wild-type levels (see Figure 1).
