# Supplementary figures and images for: First identification of proteins involved in motility of Mycoplasma gallisepticum
Source: Vet Res. 2014 Oct 17;45(1):99. doi: 10.1186/s13567-014-0099-2 (PMC4207318; doi:10.1186/s13567-014-0099-2)

## Slide 1
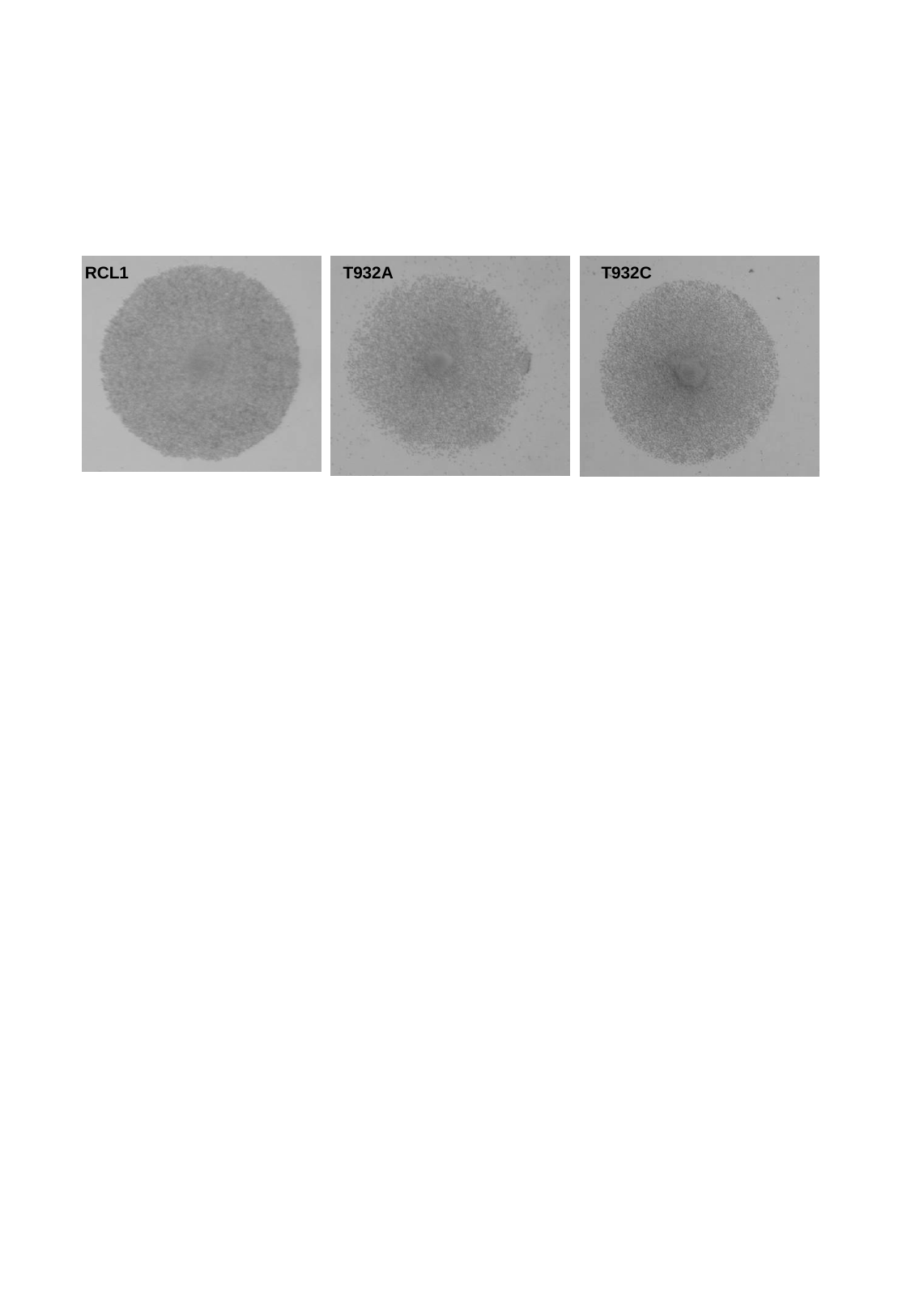

RCL1
T932A
T932C

Supplement: Additional file 1: — Qualitative HA assessment of wild-type and gliding mutants. Colonies of RCL1 and motility mutants T932A and T932C were grown on agar plates and overlaid with sheep erythrocytes. No remarkable difference in hemadsorption was seen between wild-type RCL1 and mgc2 mutant strains. [file 13567_2014_99_MOESM1_ESM.pptx]

## Slide 1
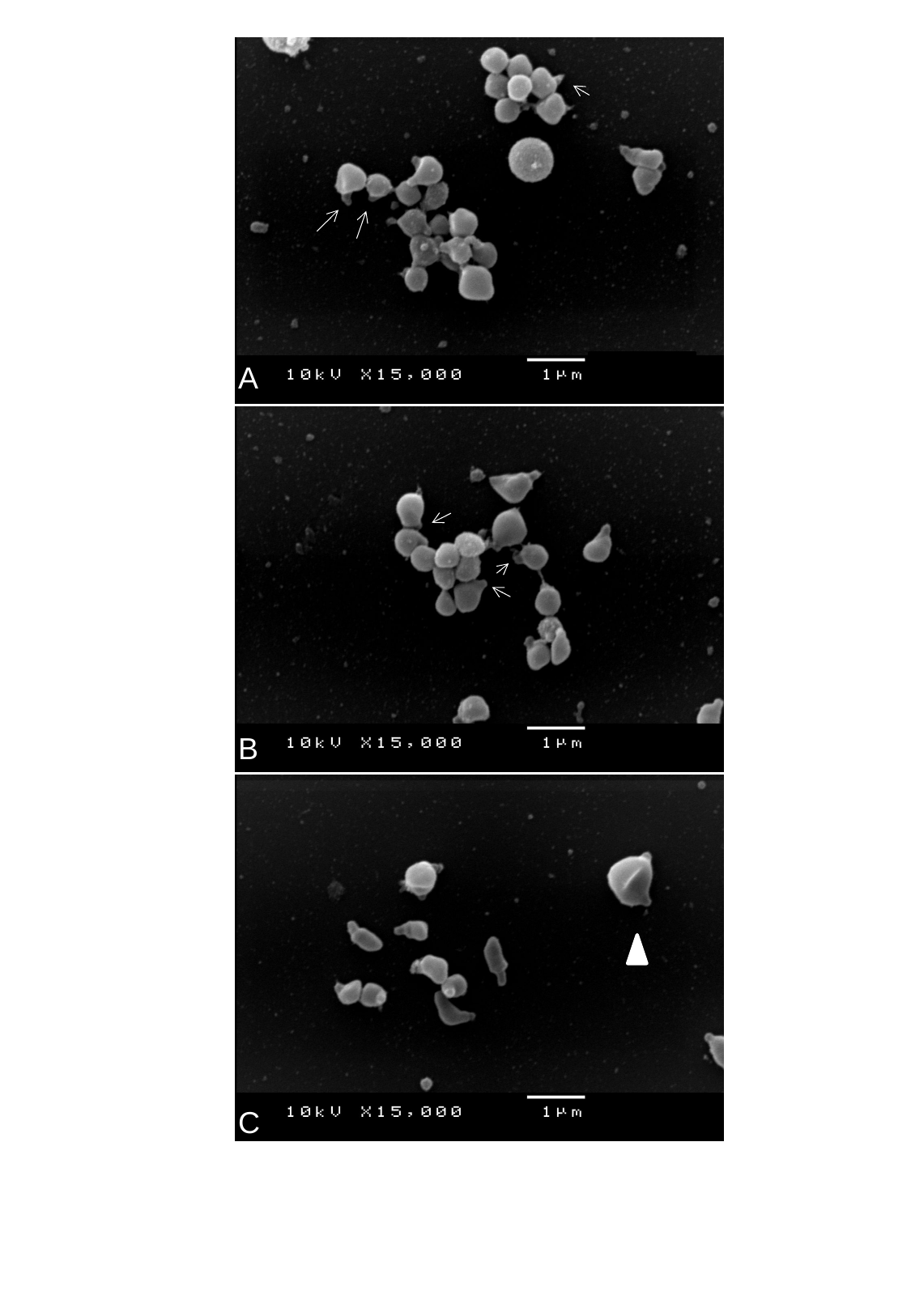

A
B
C

Supplement: Additional file 2: — SEM pictures of M. gallisepticum motility mutants. Mutant T932A (A, B) partly appeared as small spheres without a TO. In some cases TOs seemed to be placed between the glass surface and the main body of the mycoplasma cell (arrows), indicating that T932A had attached to the glass via the TO, therefore appearing spherical. Interestingly, in T932C (C) large spheroid cells with TO structures were also seen (triangle). [file 13567_2014_99_MOESM2_ESM.pptx]

## Slide 1
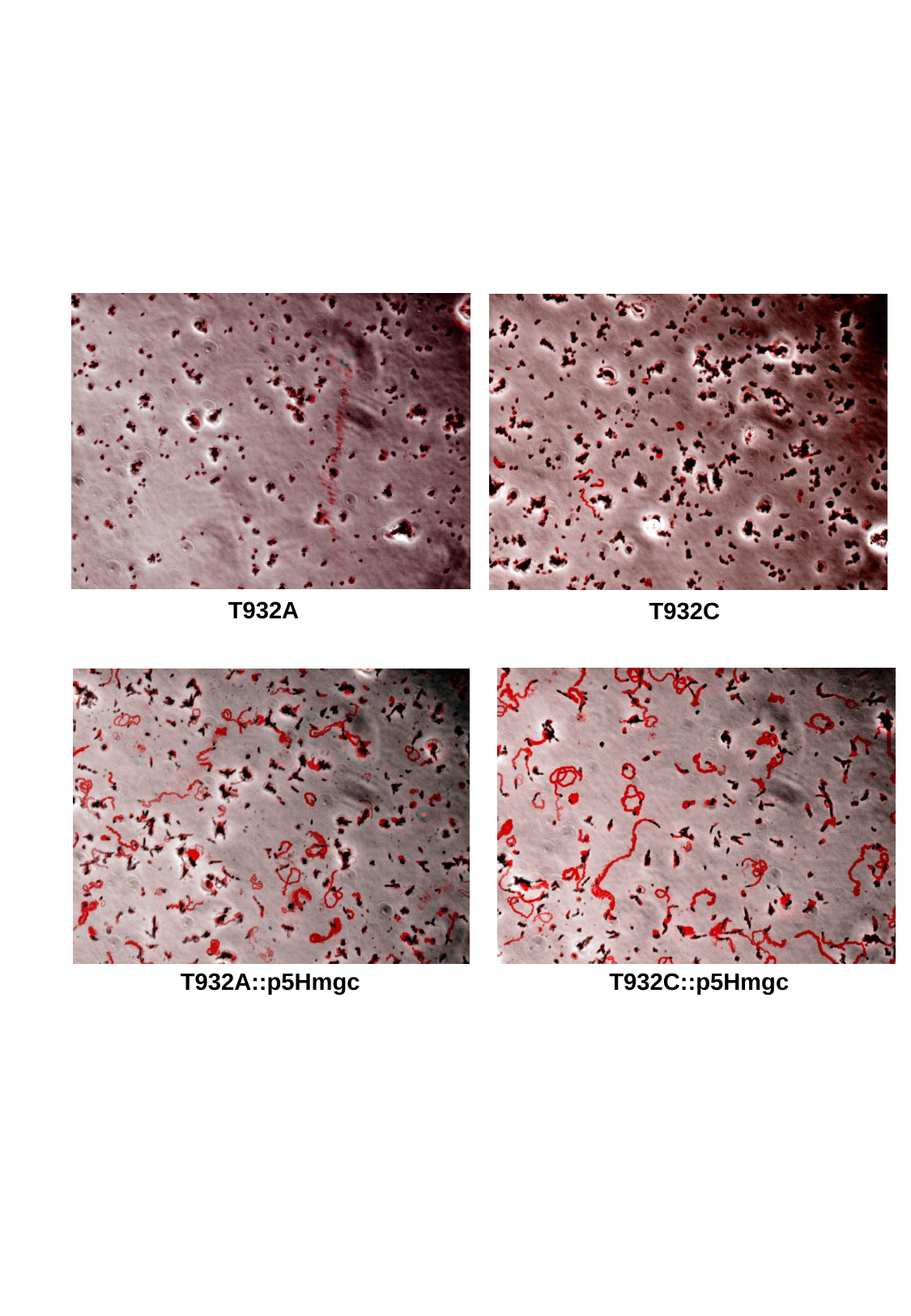

T932A
T932C
T932A::p5Hmgc
T932C::p5Hmgc

Supplement: Additional file 3: — Gliding paths of M. gallisepticum motility mutants before and after complementation. Only short gliding paths were seen, if any, in mgc2 motility mutants (T932A and T932C), while the complementation of the mutants with mgc2 (T932A::p5Hmgc and T932C::p5Hmgc) restored the gliding motility to almost wild-type levels (see Figure 1). [file 13567_2014_99_MOESM3_ESM.pptx]
